# Risk Associations between Vehicular Traffic Noise Exposure and Cardiovascular Diseases: A Residential Retrospective Cohort Study

**DOI:** 10.3390/ijerph191610034

**Published:** 2022-08-14

**Authors:** Elisa Bustaffa, Olivia Curzio, Gabriele Donzelli, Francesca Gorini, Nunzia Linzalone, Marco Redini, Fabrizio Bianchi, Fabrizio Minichilli

**Affiliations:** 1 Unit of Environmental Epidemiology and Disease Registries, Institute of Clinical Physiology, National Research Council, Via Moruzzi 1, 56124 Pisa, Italy; 2 Unit of Environmental Epidemiology and Biocomplexity Laboratory, Institute of Clinical Physiology, National Research Council, Via Moruzzi 1, 56124 Pisa, Italy; 3 Unit of Epidemiology of Rare Diseases and Congenital Anomalies, Institute of Clinical Physiology, National Research Council, Via Moruzzi 1, 56124 Pisa, Italy; 4 Municipality of Pisa, Via degli Uffizi 1, 56100 Pisa, Italy

**Keywords:** vehicular traffic noise exposure, residential cohort study, cardiovascular diseases, mortality, morbidity, hazard ratio

## Abstract

Environmental noise can induce detrimental health effects such as cardiovascular disease (CVD). The relationship between vehicular traffic noise pollution and CVD was investigated through a retrospective residential cohort study in the city of Pisa. Four exposure classes were defined for noise pollution, using noise propagation maps. The association between noise exposures and cause-specific mortality or hospitalization of the subjects of the cohort was calculated using the hazard ratio (HR) for night and day through a multiple time-dependent and sex-specific Cox regression adjusting for age, the socio-economic deprivation index, and traffic air pollution. Mortality excess for CVD and risk trends for a 1 decibel noise increment were observed among the most exposed women (mortality: HRnight_class4_ 1.15 (1.03–1.28); Trend_night_ 1.007 (1.002–1.012); HRday_class4_ 1.14 (1.02–1.27); Trend_day_ 1.008 (1.003–1.013)), particularly for ischaemic disease (mortality: Trend_night_ 1.008 (0.999–1.017); Trend_day_ 1.009 (0.999–1.018)) and cerebrovascular disease (mortality: HRnight_class3_ 1.23 (1.02–1.48), HRday_class3_ 1.24 (1.03–1.49)). Hospitalization analyses confirm mortality results. A decreased risk for hospitalization was also observed among the most exposed men (HRday_class4_ 0.94 (0.88–1.01), particularly for ischaemic disease (HRnight_class4_ 0.90 (0.80–1.02); HRday_class4_ 0.86 (0.77–0.97)) and cerebrovascular disease (HRnight_class4_ 0.89 (0.78–1.01)). Authors recommend the adoption of prevention measures aimed at mitigating noise and the activation of a monitoring of the risk profile in the Pisa population updating both the residential cohort and health data.

## 1. Introduction

Long-term exposure to environmental noise is one of the main environmental risk factors for physical and mental health and well-being. This is reflected in the seventh European Environmental Action Program (7th EEAP), which guided the European environmental policy until 2020, including actions for environmental pollution reduction, up to reaching the values recommended by the World Health Organization (WHO) [1,2]. According to the latest EEA report, air pollution and noise represent the two main environmental threats to human health, particularly in Europe, the first with an estimated impact of 400,000 premature deaths annually and the second with 12,000 premature deaths and 48,000 new cases of ischaemic heart disease (IHD) each year [3]. Exposure to noise can result in auditory and non-auditory effects. Direct damage to the auditory system may lead to hearing loss and tinnitus [4]. This is frequently caused by high noise in the workplace, loud music (continuous exposure to personal musical devices and/or frequenting of music venues), or even noisy fireworks [5]. Non-auditory health effects may occur at much lower noise levels than those causing hearing problems in the case of prolonged exposure, resulting from reactions to psychological and physiological stressful situations [6,7,8]. Moreover, effects such as stress reactions, alterations in sleep phases, and other biological and biophysical effects ranging from annoyance [9,10,11,12] to sleep disturbances [13,14], as well as hearing loss and cognitive impairment [15], endocrine effects, cardiovascular diseases, and increased incidence of diabetes may arise among people exposed to high levels of noise [6,16,17,18,19]. In addition, noise can lead to mental, behavioral, and neurological disorders that globally account for 3% of deaths and 10% of the global burden of disease [20]. More than 21 million people in Europe suffer from a high level of annoyance caused by noise, approximately 6.5 million experience sleep disturbances, and approximately 12,500 children have learning problems at school due to airport noise [21]. Based on data from six European countries, Hänninen et al. (2014) estimated that the environmental burden of disease associated with transport noise is between 400 and 1500 years of life lost due to disability or premature death per million people, making noise the second environmental disease threat in Western Europe after ambient air pollution [22].

The municipality of Pisa (Tuscany Region) is characterized by a complex structure as regards sound sources as it includes all transportation noise sources, i.e., vehicular, airport and railway traffic. There are no major ring roads and many arteries and railways stretch crossing the residential area, while the airport (national and international) is very close to the inhabited area. For these reasons, noise plays an important role in a city such as Pisa that was one of the six cities involved in the SERA project (Studio sugli Effetti del Rumore Aeroportuale—*Study on the Effects of Airport Noise*) that carried out a health impact assessment of airport noise among residents in the nearby six Italian airports. Recently, Petri et al. (2021) used data from questionnaires administered within the SERA project in order to evaluate the impact that different noise sources have on citizens’ health in terms of blood pressure and hypertension [23]. The municipal administration of Pisa has always been particularly sensitive to the issue of noise pollution and its potential effects on health, and for this reason in 2019, a retrospective residential cohort study was commissioned on the adverse health effects of noise and air pollution exposures.

This article presents the results of the residential cohort study carried out to assess the relationship between noise pollution from vehicular traffic and cardiovascular disease (CVD) among residents in Pisa considering exposure to air pollution from vehicular traffic and socioeconomic factors.

## 2. Materials and Methods

This study was designed in 2019 as part of an agreement between the municipality of Pisa and the Institute of Clinical Physiology of the National Research Council of Italy, IFC-CNR (Unit of Environmental Epidemiology and Disease Registries). The study was carried out in accordance with the Helsinki Declaration of Ethical Principles. No personal identifiers were sent to the research staff, all addresses were geocoded and the personal data were analyzed anonymously.

### 2.1. Study Design

#### 2.1.1. The Cohort Study

This retrospective study evaluated the health risk in a cohort of residents in the municipality of Pisa over the follow-up period (see Section 2.1.2) in relation to exposure to both noise and air pollution, considering (i) age and sex as individual risk factors and (ii) a proxy of socioeconomic status at the census area of residence.

#### 2.1.2. Definition of the Cohort and the Follow-Up Period

The study cohort included the total of inhabitants residing in Pisa (domain of the study) for at least one year during the follow-up period (2001–2012 for mortality, 2001–2014 for hospitalization). Demographic and residential data of all residents were provided by the General Registry Offices of Pisa. The cohort is open and dynamic, i.e., the permanence period of a subject within the cohort (person years) starts from the beginning to the end of the follow-up, or sub-periods for those born, dead, or, if resident, immigrants or emigrants in the period. With regard to hospital admissions, for each cause of hospitalization, the calculation of person years concerns the period from the entry into the cohort until the first admission for that cause. In essence, the cohorts built are the same number of the causes of hospitalization considered. In case of change of residence, the total of person years is the sum of person years calculated for the permanence at each address. Resident subjects died or hospitalized outside the study area but within the study period are considered in the same way as those who died/hospitalized within the study area. For all subjects the residential addresses were georeferenced (assignment of geographical coordinates to each address).

### 2.2. Exposure Assessment

#### 2.2.1. Exposure to Noise

**Figure 1:**
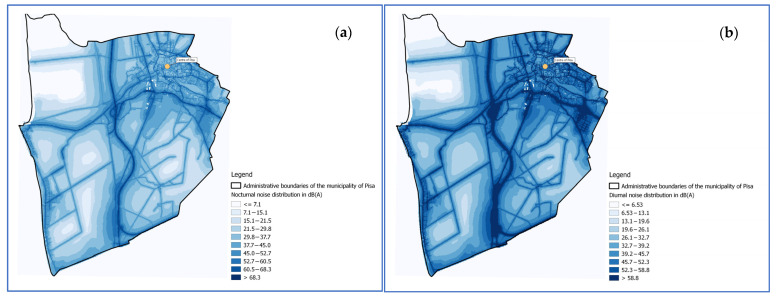
Noise propagation map during the night period (22:00–06:00) (**a**) and daytime period (06:00–22:00) (**b**) within the administrative limits of Pisa’s municipality.

The vehicular traffic noise propagation maps were created using a model based on the ray tracing method that allows acoustic modelling in accordance with the nationally and internationally standards adopted. Modeling was done using SoundPLAN 8.1 software (SoundPLAN GmbH, Backnang, Germany). For each source, the acoustic maps of the A-weighted long-term equivalent continuous levels determined during the day period (interval 06:00–22:00; L_D_) (Figure 1a) and night period (interval 22:00–06:00; L_N_) were created (Figure 1b), using the indicators of the current Italian legislation (Framework Law n.°447/95).

**Figure 2:**
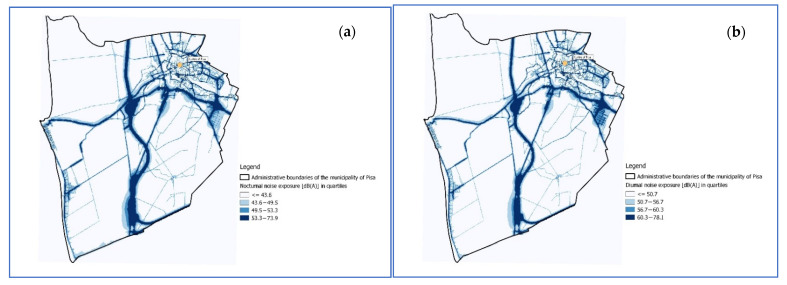
Representation of the quartiles of the distribution of nocturnal (**a**) and diurnal (**b**) noise exposure.

Since direct individual measurements were not available, proxy of noise exposure of each subject in the cohort was defined by attributing the noise pollution value estimated by the noise propagation models to the residential address. Using the noise map, for each georeferenced subject the history of noise exposure was estimated, considering the residence address changes within the area during the follow-up period. Subsequently, each individual belonging to the residential cohort was assigned one of the 4 levels of noise exposure, defined on the basis of the quartiles of the distribution of the noise values attributed to the civic buildings. The 4 exposure classes were defined as follows (Figure 2):

- Class 1 (reference): nighttime <43.7 dB (A); daytime; <50.7 dB (A);
- Class 2: nighttime 43.7–49.5 dB (A); daytime 50.7–56.7 dB (A);
- Class 3: nighttime 49.5–53.3 dB (A); daytime 56.7–60.3 dB (A);
- Class 4: nighttime 53.3–73.9 dB (A); daytime 60.3–78.1 dB (A).

#### 2.2.2. Exposure to Vehicular Traffic Air Pollution

**Figure 3:**
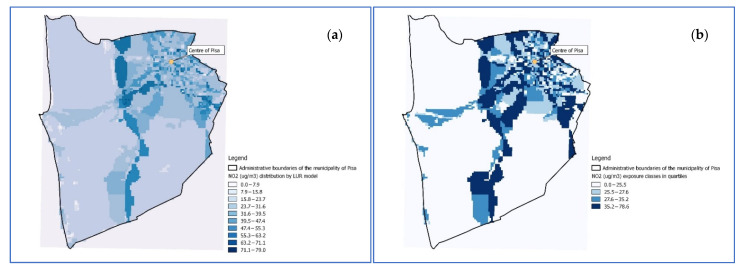
Diffusion model (**a**) and representation by quartiles (**b**) of the nitrogen dioxide (NO_2_) as proxy of vehicular traffic exposure.

Proxy of individual exposure of vehicular traffic pollution, considering nitrogen dioxide (NO_2_) as atmospheric pollution surrogate, were obtained based on the data and method used in a previous study conducted in Pisa [24], by a Land Use Regression model estimated in the setting of the *European Study of the Cohorts of Air Pollution Effects* (ESCAPE) project [25] (Figure 3a).

Each cohort subject was assigned one of the 4 levels of vehicular traffic exposure (defined on the basis of the quartiles of NO_2_ levels distribution) overlapping the map of georeferenced civic buildings with the Land Use Regression model. The 4 exposure classes were defined as follows (Figure 3b):

- Class 1 (reference): <25.5 µg/m^3^;
- Class 2: 25.5–27.6 µg/m^3^;
- Class 3: 27.6–35.2 µg/m^3^;
- Class 4: 35.2–78.6 µg/m^3^.

#### 2.2.3. Exposure to Socio-Economic Factors

The socio-economic status is reported by the literature as associated with both health outcomes and environmental factors and was considered as a confounder of the relationship under study. The indirect measurement of socioeconomic factors is usually defined through a socioeconomic deprivation indicator (ID) calculated at the census section level using variables from the census of the population (ISTAT sources) [26]. The ID was calculated on the basis of 5 variables of the 2011 population census:

- Percentage of the population with an education level equal to or lower than the elementary school certificate (failure to reach compulsory schooling);
- Percentage of the active population unemployed or seeking their first job;
- Percentage of occupied rented dwellings;
- Percentage of single-parent families with cohabiting dependent children;
- Population density (number of occupants per 100 m^2^).

**Figure 4:**
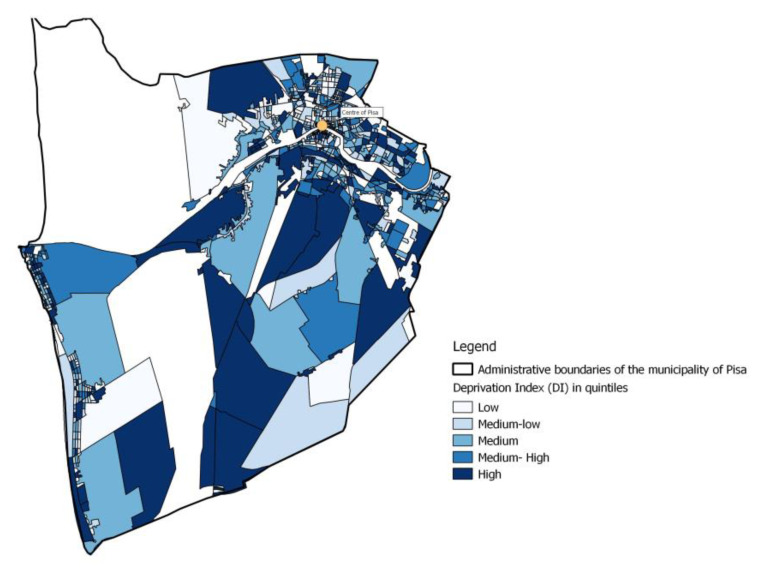
Socio-economic categorical deprivation index (DI) distribution by census sections of the municipality of Pisa.

The ID is a continuous variable and represents the distribution of the differences in socioeconomic deprivation of each census section of the study area compared to the regional average deprivation. Individual socioeconomic deprivation index (DI) was estimated by overlaying the georeferenced cohort layer with the map of socioeconomic DI calculated at the census block level [26]. Five categories in the total population of DI distribution were defined, from the least (light blue) to the most (dark blue) deprived through the quintiles of DI (Figure 4).

### 2.3. Health Indicators

Mortality data for the period 2001–2012 were obtained from the Regional Mortality Registry, while the hospital admission data for the period 2001–2014 were derived from the Regional Hospital Discharge Records, including the extra-regional mobility.

As regards hospital admissions (proxy of incident cases), only the first admission during the follow-up period was selected for each cause of hospitalization considered.

The causes of mortality and hospital admission were selected from those that the scientific literature considers being most persuasively associated with noise exposure, namely:

- Diseases of the circulatory system (CVD) (International Classification of Diseases IX revision ICD-IX Code–390–495);
- Hypertensive diseases (ICD-IX Code–401–405);
- Ischaemic heart diseases (IHD) (ICD-IX Code–410–414);
- Acute myocardial infarction (AMI) (ICD-IX Code–410);
- Cerebrovascular diseases (ICD-IX Code–430–438);
- Stroke (ICD-IX Code–434, 435, 437, 446).

Mortality due to hypertension and stroke was not considered due to the scarce number of events. Furthermore, as pharmaceutical prescriptions were not available, we used the hospital discharge records to evaluate the occurrence of hypertensive diseases; therefore, in the section of hospitalization analyses, comments on this outcome were reported for purely descriptive purposes.

### 2.4. Statistical Analyses

The relationship between each outcome of mortality/morbidity and exposure to noise pollution from vehicular traffic was estimated by the hazard ratio (HR) with a confidence interval of 95% of probability (95% CI). HR was calculated by multiple time-dependent sex-specific Cox regression models, comparing more exposed areas to the less exposed area (considered as reference) and adjusting for the age class at diagnosis, DI class, and exposure class to NO_2_.

An adjusted linear trend of risk (trend) for 1 decibel noise increment with the relative 95% CI and *p*-value was also calculated using the proxy of individual exposure of noise pollution. Analyses of sensitivity were also performed through three different models considering the aforementioned adjusting factors separately. Specifically, age was used in model 1, age and DI in model 2, and age and NO_2_ exposure in model 3.

The threshold for statistical significance was set at *p* < 0.05, but associations with *p* < 0.10 were also reported, to reason more broadly than exclusively considering the pure concept of statistical significance [27]. Schoenfeld’s test to evaluate the proportional hazard assumption in all Cox regression models was also carried out.

Analyses were performed for the full general population and separately for men and women using STATA v.13 (StataCorp. 2013. Stata Statistical Software: Release 13. StataCorp LP, College station, TX, USA).

## 3. Results

Table 1 and Table 2 show descriptive cohort characteristics (sex, age, socioeconomic status, noise exposure, and NO_2_ exposure from vehicular traffic) from mortality and hospitalization data.

Table 3 and Table 4 show the relationships between the exposure to nocturnal and diurnal noise from vehicular traffic and the mortality/hospitalization causes for the subjects, considering 4 classes of noise exposure (from high, class 4, to low, class 1, as reference) for the general population (men and women, *M* + *W*) and separately by sex. The risk trend analysis for each decibel of noise increase is also reported. To highlight the results for the most exposed classes, only statistically significant results or those at the limits of statistical significance for classes with the highest noise levels (classes 3 and 4) referred to the lowest noise class 1, were commented.

Results of sensitivity analyses are comparable to those obtained from the analyses carried out with the model that considered all the adjustment factors, of which results are reported below.

### 3.1. Results of Statistical Analyses

#### 3.1.1. Descriptive Analysis—Mortality

Considering the characteristics of the cohort through the mortality data in the period 2001–2012 (Table 1), the cohort counted 132,293 subjects and 985,022 person-years, 52.6% women of which 24.5% were aged over 64 years. The mortality rate for CVD was higher among women and significantly increased with increasing age classes (Table 1). As the exposure class to traffic noise increased, the risk of CVD mortality increased (Table 1). In the higher deprivation class, the rate was slightly higher than in the other deprivation classes. As both night and day noise exposure increased, the mortality rate from CVD increased. No increase in mortality rate was observed for increasing classes of NO_2_ exposure from vehicular traffic (Table 1).

#### 3.1.2. Descriptive Analysis—Hospital Admission

As for hospitalization, in the period 2001–2014, the cohort included 139,710 subjects and 1,107,023 person-years. A total of 53.1% of the total were women, of which 21.9% were aged over 64 years. The hospitalization rate for CVD was higher among men and significantly increased with the age classes (Table 2). In the higher classes of exposure to vehicular traffic noise, the risk of hospitalization is higher than the reference (Table 2). In the higher deprivation class, the hospitalization rate was slightly higher compared with other deprivation classes. No increases in hospitalization rate were observed as the NO_2_ exposure from vehicular traffic increased (Table 2).

#### 3.1.3. Cardiovascular Disease and Noise Exposure—Mortality Analysis

Considering CVD among the general population, the results showed an increased risk at the limit of statistical significance for exposure to medium noise levels of 8% during both night and day and a statistically significant risk excess of 11% for the night period and of 10% during the day for those exposed to higher noise levels (Table 3). Furthermore, a linear trend of increasing risk association with increasing exposure to both nighttime (+0.6%) and daytime (+0.6%) noise was found (Table 3). A 15% statistically significant increase in risk was observed among women during the nocturnal period for medium and high noise exposure, and 14% for exposure to medium and high noise levels during the day (Table 3). In addition, among women, a linear trend of increasing the CVD risk association of 0.7 and 0.8% for 1 dB increments in night and day noise exposure was observed (Table 3).

Considering the sub-causes, increasing trends close to statistical significance emerged for IHD among women exposed to both night (+0.8%) and day (+0.9%) noise (Table 3).

Furthermore, a statistically significant excess risk of cerebrovascular disease by 23% at night and 24% during the day is reported for women exposed to medium levels of noise exposure (Table 3).

#### 3.1.4. Cardiovascular Disease and Noise Exposure—Hospital Admission Analysis

Regarding hospitalization for CVD, significant results emerged for the upper class of noise exposure, although they differed by sex. Among women, statistically significant risk increases of 8% during both night and day were observed (Table 4). A linear trend analysis showed among women a 0.5% increase in CVD risk for night noise and a 0.4% increase for daytime noise (Table 4). During the day, a risk defect of 6% was observed among men, even if not statistically significant (Table 4).

As regards IHD, a defect of 10% in the upper class of night noise exposure, although at the limit of the statistical significance, was reported among men alone. In the same class of noise exposure, in the diurnal period, risk defects were observed of 8% for both sexes (although at the limit of statistical significance) and of 14% among men (statistically significant) (Table 4).

During the night period, an excess at the limit of statistical significance of 21% was reported for acute myocardial infarction (AMI) among women belonging to class 4 of noise exposure. In addition, the trend analysis showed a 0.9% risk increase for both nocturnal and daytime noise increases (Table 4).

For cerebrovascular disease, a risk defect at the limit of statistical significance of 11% was observed only among men during the daytime for the higher class of noise exposure (Table 4). Furthermore, the trend analysis showed a 0.5% risk increased for nocturnal noise increases only among women (Table 4).

Table 5 summarizes the results of the statistical analyses in a descriptive way, to facilitate the reading of the results.

## 4. Discussion

The aim of this research was to assess the relationship between noise exposure from vehicular traffic and CVD onset, considering the potentially correlated factors that could have a confounding role, also including the exposure to air pollution from vehicular traffic.

Considering both night and day noise exposure, the results showed risk mortality excesses for (i) CVD among the general population and women belonging to both medium and high class of noise exposure and (ii) cerebrovascular diseases among women in the medium noise exposure class. Regarding hospitalization, for both night and day exposure, increased risks in association with increased noise were found only among women considering CVD and AMI; in contrast, only for daily exposures decreased risks for CVD, IHD, and cerebrovascular disease were reported among only men in the higher class of noise exposures. Our findings on mortality excesses for CVD are consistent with a Swiss population study and a cohort study in Copenhagen, both reporting a positive association between CVD mortality and noise from vehicular traffic [28,29,30]. Differently, a recent study performed on three cohorts found an association between noise exposure and CVD incidence only for the second quintile of L_DEN_ (52.3–54.0 dB (A)), which disappeared after adjustment for classical risk factors (type of occupation, education level, and cigarette smoking) and air pollution (particulate matter with a diameter of 10 µm (PM_10_) and NO_2_) [31].

The results available on specific subgroups of CVD are persuasive; therefore, we discussed them separately in the following paragraphs.

As previously clarified (Section 2.3), in this study, hypertensive diseases were evaluated by hospital discharge records instead of pharmaceutical prescriptions, probably resulting in an underestimated association. Therefore, the comments hereinafter on this outcome are reported for purely descriptive purposes. Unlike our study, previous studies documented evidence of positive and significant associations between noise exposure and hypertension [32,33], especially among men during the night [34,35]. A recent investigation conducted in Pisa also found a more relevant exposure–response association for hypertensive risk among men, although this study analysed a larger number of noise sources all together (traffic, railway, aircraft, and recreational noise) [23]. In agreement with our results, a case–control study performed on subjects residing in the region around the Frankfurt airport found no associations with any of the traffic noise sources [36].

Considering both nocturnal and diurnal periods of noise exposure, an excess risk of mortality for IHD was observed among women (an excess was also found in the hospitalization analysis though this result did not reach statistical significance), while risk defects were reported among men most exposed to noise. The results on the association between road traffic noise and IHD are inconsistent. Some studies reported positive associations [28,37,38,39,40,41,42,43,44], whereas in other studies no associations were found [31,34,45,46,47,48,49,50]. Our findings of increasing mortality trends close to statistical significance for IHD among women exposed to both night and daytime noise are in line with the study of Piko et al., who reported an increased risk of IHD in women exposed to road traffic [51]. Other studies showed an association between IHD mortality and road traffic noise [28,30] or between diurnal noise exposure and mortality, but not with hospitalization for IHD [40]. The difference in results between men and women could be due to residual confounding, attributable to both biological and environmental factors [52]. To the best of our knowledge, the present study is the first one reporting negative associations among men between traffic noise exposure and IHD hospitalization.

Noteworthy, an excess risk of hospitalization for AMI was shown among women in class 4 only in the night period, and a risk trend both for daytime and nighttime noise. In the literature, some findings on the associations between AMI and road traffic noise can be found in the general population considering both men and women, adjusting for air pollution, socioeconomic status, and factors related to lifestyle [28,42,50,53], confirming that road traffic noise and air pollution are correlated. Other studies revealed an increase in AMI risk only in men [34,54,55], contrary to what we observed only among women. Only Bodin et al. (2016) observed that the incidence ratio did not increase for AMI, even after considering other individual confounding factors (age, gender, body mass index, smoking habits, education level, alcohol consumption, civil status, year, country of birth, and physical activity) [48].

Excesses of mortality risk for cerebrovascular disease were reported among women in the third class of noise exposure. Conversely, hospitalization analyses highlighted risk reductions among only men. Few studies explored this association and with conflicting results. Two studies found that a high noise level, specifically when the noise increased by 10 dB (A), significantly increased the hospitalizations and the mortality rate for cerebrovascular disease [56,57]. Other studies did not find a significant increased risk of hospital admissions [58] or associations between the annual mean level of L_D_ and the incidence of cerebrovascular diseases [31].

Research analyzing road traffic effects on stroke is currently scarce and shows inconsistent results. In the present study, no association between noise exposure and stroke was revealed, independently from the adjusting factor, and in accordance with a large number of studies performed in the Netherlands, Sweden, and Germany [31,49,51,59]. Other authors found that road traffic noise increased the risk of stroke independently from other factors (NO_x_ air concentration, smoking habits, diet, and alcohol consumption) [28,60,61,62,63]. Cole-Hunter et al. (2021) reported a positive association between long-term exposure to road traffic noise and the risk of overall stroke, but without adjusting for air pollution (PM_2.5_ and NO_2_) [64]. Furthermore, positive, but not statistically significant, associations with mortality for cardiovascular and ischemic cardiopathy and stroke in both adults and the elderly were observed [40].

Actions to reduce noise exposure would result in limited annoyance, an improvement in children’s school performance, in sleep quality, and a lower prevalence of circulatory system diseases. Our results, consistent with the most recent studies, indicate that an effort by environmental policies aimed at lowering noise levels exposure in urbanized and industrial areas is indispensable. Some proposed mitigation strategies to improve public health include reducing noise at the source, active noise control, optimized traffic operations, better infrastructure planning, and better acoustic isolation in situations where other options are not feasible and adequate [65]. In fact, although the efforts of biomedical sciences are mainly directed toward diagnosis, treatment, and prevention of the traditional cardiovascular risk factors (i.e., diabetes, smoking, arterial hypertension, and hyperlipidemia) [66], recent evidence indicated that even risk factors present in the physical environment could facilitate the development of CVD [66]. In the last few decades, several studies have reported associations between noise (from vehicular, air, and rail traffic) and increased risk of CVD [65,67,68]. Already in 2011, Babisch (2014) posed the following question: “*The question at present is no longer whether noise causes cardiovascular effects*”, thus placing the role of noise in the onset of CVD as a fixed point, “*it is rather: what is the magnitude of the effect in terms of exposure-response relationship (slope) and the onset or possible threshold (intercept) of the increase in risk?*” [37]. Babisch, clearly leaned towards an etiological role of traffic noise in causing adverse health effects through both direct (e.g., sleep disturbances) and indirect (e.g., annoyance) modalities [37]. The general mechanism of the acute response to noise is based on the release of stress hormones (adrenaline and cortisol), through the activation of the hypothalamus–pituitary–adrenal axis, and the sympathetic–adrenal–medullary axis [57,68]. In the long term, these adaptive physiological responses can cause adverse pathophysiological alterations that can contribute to the onset of CVD [69]. In the last decade, in addition to the results obtained on annoyance and sleep disorders, the basis of evidence related to the cardiovascular effects of long-term exposure to traffic noise, in particular that deriving from transport sources on roads, has been strengthened. Of note, to date, most of the evidence correlated noise exposure to hypertension [33] and IHD [37,44], although for the latter, further studies are warranted in order to reinforce the comprehension of the dose–response relationship and identify susceptible subgroups. In addition, potential associations with other cardiovascular outcomes such as stroke [40,62], atherosclerosis [70], atrial fibrillation [71], cardiac arrest [28,72], and arterial stiffness [73] have been recently documented and need to be confirmed.

The strength of this study is the residential cohort design, one of the most advanced approaches adopted by other national environmental epidemiology studies [74,75,76], in which individual exposure at the residence was assessed by noise dispersion models using socioeconomic status and other environmental sources of pollution as potential confounders. It also allows to associate each subject’s health history with noise exposure during the study period, thoroughly considering individual migration movements (external and internal) in the study area. Furthermore, the assignment of exposure to each individual based on a diffusion model reduces the arbitrariness of subjective choices.

Our study has some limitations concerning the evaluation of the exposure to (i) noise and air pollution, based on the assignment of residence address without considering daily shifts of each subject as for example the workplace and the kind of job, (ii) noise level considering only the noise propagation model, (iii) NO_2_ level estimated through a land use regression model, and (iv) the difference in the detail of the exposure assessment: the comparison between the road traffic noise map and the air pollution map shows that the NO_2_ map has a more crude exposure estimation with, as a consequence, a higher risk of misclassification. Keeping into account these limitations, bias in individual risk estimation cannot be ruled out. In addition, the retrospective cohort design adopted did not allow us to consider individual risk factors associated with circulatory system diseases, such as work history, exposure to tobacco smoke and alcohol consumption, physical activity, body mass index, level of education, family history for CVD, and other lifestyle habits. In the face of these shortcomings, it is reasonable to argue that the use of the DI may have reduced the effect of these possible confounding factors, as it is recognized as a reliable proxy of individual factors [26].

Finally, it should be noted that multiple tests were not performed as the analysis was not exploratory but considered only those diseases recognized as associated with noise pollution. In evaluating the results, the authors believe that it is fundamental not to rely exclusively on statistical significance but to consider mainly epidemiological and biological plausibility.

## 5. Conclusions

In recent years, several harmful health effects associated with the exposure to environmental noise have been documented. Noise exposure can indirectly induce stress that can promote psychological symptoms and disorders which, in turn, are associated with brain and cardiovascular dysfunction.

Our study found excess mortality risks for CVD among the general population and among women belonging to medium-high and high noise exposure classes and for cerebrovascular and ischemic diseases only among women with a medium and a high exposure to noise, respectively. Increased hospitalization risks for CVD (particularly for cerebrovascular and acute myocardial infarction) were observed only among the more exposed women. A decreased risk for hospitalization was also observed among the most exposed men, particularly for IHD and cerebrovascular disease. Therefore, it remains unclear if the observed effects result from differences in sex-related biology, gender-related environmental factors, or from a combination of both. The presence of a significant risk trend strengthens the hypothesis of a risk association between CVD and noise exposure. Overall, despite the methodological limitations, the present study shows signals supporting the relationship between noise and cardiovascular disorders and it shows a significant increase in cardiovascular risk for the most exposed subjects.

The findings of the present study reinforce the knowledge of the role of noise exposure as a risk factor for CVD. Considering that more than 125 million people in Europe are exposed to vehicular traffic noise >55 dB (A), as recently reported by the EEA, it follows that there is a public health concern and an urgent need to promote environmental measures that are proven to reduce noise exposure. The European Union has established guidelines to mitigate the negative consequences of chronic exposure to environmental noise including noise reduction at the source, active noise control, optimized traffic operations, improved infrastructure planning, and noise insulation in environments where other options are not feasible and appropriate. Mitigation policies could limit annoyance, improve children’s school performance, sleep quality, and decrease the occurrence of circulatory system diseases. Therefore, the relevance of epidemiological studies in the urban context is emphasized by the actions undertaken as a consequence of the awareness gained by decision makers on the basis of such epidemiological evidence. Pisa is a medium-sized city with a high population density, located in a European high-income country where anthropogenic pressures are responsible for a deterioration in citizens’ health. The present study does not contain specific urban sustainability actions, but it is strongly evocative and advocating actions to promote public health. The city administration has recently showed sensitivity to the noise issue through the development of several programmatic and strategic tools [77]. These tools are subject to consultation and participatory reviews to identify specific paths of noise reduction and mitigation (including the strengthening of the intramodality of transport through tramways among wide areas and the location of exchanger parking areas). This study, despite all its limitations, represents another piece of the current scientific literature providing the necessary weight to these actions in order to carry out their fulfilment. In particular, public opinion can endorse transformative urban planning interventions if scientific evidence supports the urgency. To move the city towards noise reduction is therefore necessary to extend knowledge and discussion to the audience of interested parties, evaluating the alternatives according to already applied methods and with win-win results for the involved parties [78].

To apply scientific knowledge transfer of the findings into decision-making it is worth considering the following suggestions:

- Define technical actions within teams of epidemiologists, urban developers, environmental practitioners, administrators, and representatives of the city neighborhoods. A transfer of the present scientific evidence into local decisions should adopt a transparent and layman language;
- Preserve the role of research as an independent source of information and objectively adopting the best scientific approach to provide and interpret results. A “third-party” contribution can move decisions to a greater acceptance;
- Set a permanent observatory for monitoring policy interventions at the city level. The assessment of the impacts of adopted or planned strategies is the best way to add value to research findings and improve the health indicators over time.

These suggestions may stimulate further improvement in exposure and health outcomes at the individual level also considering the individual risk characteristics such as behavior and lifestyle habits.

In conclusion, the setting of the urban area of Pisa supports the scientific evidence that environmental noise is a not a negligible risk factor for cardiovascular health, therefore continuous monitoring of the population is recommended also through the administration of questionnaires to improve the evaluation of the population exposure and health policy actions deemed effective to reduce the levels of exposure to noise pollution.

## Figures and Tables

**Table 1:** Descriptive cohort characteristics by risk factors–mortality data (period 2001–2012).

Cohort		Person-Years	Number of Deaths for CVD	Crude Rate × 1000 Person-Years	95% CI	
	Total	985,022	4854	4.93	4.79	5.07
**Sex**	Women	518,783	2870	5.53	5.33	5.74
	Men	466,239	1984	4.26	4.07	4.45
**Age classes** **(years)**	0–44	476,355	40	0.08	0.06	0.11
	45–54	131,925	73	0.55	0.44	0.70
	55–64	135,510	191	1.41	1.22	1.62
	65–74	120,543	648	5.38	4.98	5.81
	75–84	91,683	1942	21.18	20.26	22.15
	85+	29,006	1960	67.57	64.65	70.63
**Classes of socio-economic deprivation**	Low	193,035	910	4.71	4.42	5.03
	Medium-low	195,292	904	4.63	4.34	4.94
	Medium	201,576	992	4.92	4.62	5.24
	Medium-high	196,028	948	4.84	4.54	5.15
	High	193,552	1062	5.49	5.17	5.83
**Classes of exposure to nocturnal noise (dB(A))**	Class 1 (reference): <43.7	245,342	1074	4.38	4.12	4.65
	Class 2: 43.7–49.5	252,567	1216	4.81	4.55	5.09
	Class 3: 49.5–53.30	247,869	1249	5.04	4.77	5.33
	Class 4: 53.3–73.9	239,220	1315	5.50	5.21	5.80
**Classes of exposure to diurnal noise (dB(A))**	Class 1 (reference): <50.7	245,621	1082	4.41	4.15	4.68
	Class 2: 50.7–56.7	250,101	1187	4.75	4.48	5.02
	Class 3: 56.7–60.3	249,401	1246	5.00	4.73	5.28
	Class 4:60.3–78.1	239,875	1339	5.58	5.29	5.89
**Classes of exposure to NO_2_ (µg/m^3^)**	Class 1 (reference): <25.5	210,071	912	4.34	4.07	4.63
	Class 2: 25.5–27.6	215,578	1104	5.12	4.83	5.43
	Class 3: 27.6–35.2	302,240	1484	4.91	4.67	5.17
	Class 4: 35.2–78.6	256,716	1354	5.27	5.00	5.56

Notes: CVD: cardiovascular diseases; 95% CI: confidence interval at 95% of probability; NO_2_: nitrogen dioxide.

**Table 2:** Descriptive cohort characteristics by risk factors–hospitalization data (period 2001–2014).

Cohort		Person-Years	Number of Hospitalizations for CVD	Crude Rate × 1000 Person-Year	95% CI	
**Sex**	Total	1,107,023	13,277	11.99	11.79	12.20
	Women	588,219	6257	10.64	10.38	10.90
	Men	518,804	7020	13.53	13.22	13.85
**Age classes** **(years)**	0–44	557,381	927	1.66	1.56	1.77
	45–54	156,370	1061	6.79	6.39	7.21
	55–64	150,762	2250	14.92	14.32	15.55
	65–74	128,187	3519	27.45	26.56	28.37
	75–84	87,768	4099	46.70	45.30	48.16
	85+	26,555	1421	53.51	50.80	56.37
**Classes of socio-economic deprivation**	Low	216,732	2501	11.54	11.10	12.00
	Medium-low	220,026	2530	11.50	11.06	11.96
	Medium	226,222	2639	11.67	11.23	12.12
	Medium-high	221,501	2624	11.85	11.40	12.31
	High	216,034	2931	13.57	13.09	14.07
**Classes of exposure to nocturnal noise (dB(A))**	Class 1 (reference): <43.7	276,582	3181	11.50	11.11	11.91
	Class 2: 43.7–49.5	284,178	3372	11.87	11.47	12.27
	Class 3: 49.5–53.3	279,133	3388	12.14	11.74	12.55
	Class 4: 53.3–73.9	267,106	3336	12.49	12.07	12.92
**Classes of exposure to diurnal noise (dB(A))**	Class 1 (reference): <50.7	276,849	3181	11.49	11.10	11.90
	Class 2: 50.7–56.7	280,855	3289	11.71	11.32	12.12
	Class 3: 56.7–60.3	280,787	3473	12.37	11.96	12.79
	Class 4:60.3–78.1	268,508	3334	12.42	12.00	12.85
**Classes of exposure to NO_x_ (µg/m^3^)**	Class 1 (reference): <25.0	237,754	2714	11.42	10.99	11.85
	Class 2: 25.5–27.6	240,157	3106	12.93	12.49	13.40
	Class 3: 27.6–35.2	339,832	4032	11.97	11.50	23.24
	Class 4: 35.2–78.6	288,787	3423	11.85	11.46	12.26

Notes: CVD: cardiovascular diseases; 95%CI: confidence interval at 95% of probability; NO_2_: nitrogen dioxide.

**Table 3:** Mortality analysis (period 2001–2012) by period (nocturnal/diurnal), class of noise exposure, and gender.

Cause (Code ICD-IX)	Gender	Class Exp.	Nocturnal Period									Diurnal Period								
			*n*	HR	*p*	95% CI		Trend	*p*	95% CI		*n*	HR	*p*	95% CI		Trend	*p*	95% CI	
***Diseases of the Circulatory System*** ***(390–459)-CVD***	***M + W***	***2***	1216	1.03	0.473	0.95	1.12					1187	1.02	0.693	0.94	1.11				
		***3***	1249	1.08	0.061	1.00	1.17					1246	1.08	0.079	0.99	1.17				
		***4***	1315	1.11	0.015	1.02	1.20	1.006	0.003	1.002	1.010	1339	1.10	0.029	1.01	1.19	1.006	0.003	1.002	1.010
	***M***	***2***	463	0.91	0.171	0.80	1.04					452	0.89	0.068	0.78	1.01				
		***3***	505	0.99	0.931	0.88	1.13					507	0.99	0.902	0.88	1.12				
		***4***	541	1.07	0.313	0.94	1.21	1.004	0.207	0.998	1.010	547	1.05	0.456	0.93	1.19	1.004	0.250	0.997	1.010
	***W***	***2***	753	1.12	0.041	1.00	1.25					735	1.12	0.043	1.00	1.25				
		***3***	744	1.15	0.010	1.04	1.28					739	1.14	0.015	1.03	1.27				
		***4***	774	1.15	0.013	1.03	1.28	1.007	0.004	1.002	1.012	792	1.14	0.018	1.02	1.27	1.008	0.003	1.003	1.013
***Ischaemic Heart Diseases*** ***(410–414)-IHD***	***M + W***	***2***	396	0.95	0.441	0.82	1.09					388	0.93	0.293	0.80	1.07				
		***3***	424	1.03	0.702	0.89	1.18					412	0.98	0.765	0.85	1.12				
		***4***	455	1.09	0.248	0.95	1.25	1.005	0.134	0.998	1.012	467	1.07	0.319	0.94	1.23	1.005	0.170	0.998	1.011
	***M***	***2***	176	0.82	0.048	0.67	1.00					178	0.82	0.052	0.67	1.00				
		***3***	208	0.96	0.689	0.79	1.17					197	0.90	0.275	0.74	1.09				
		***4***	215	1.01	0.894	0.83	1.23	1.002	0.672	0.993	1.011	222	1.02	0.868	0.84	1.23	1.001	0.858	0.991	1.010
	***W***	***2***	220	1.09	0.385	0.90	1.33					210	1.04	0.670	0.86	1.27				
		***3***	216	1.11	0.322	0.91	1.35					215	1.07	0.493	0.88	1.30				
		***4***	240	1.18	0.104	0.97	1.43	1.008	0.078	0.999	1.017	245	1.14	0.176	0.94	1.39	1.009	0.068	0.999	1.018
***Acute Myocardial Infarction*** ***(410)-AMI***	***M + W***	***2***	147	0.97	0.813	0.77	1.23					143	0.95	0.642	0.75	1.19				
		***3***	152	1.00	0.981	0.80	1.26					147	0.95	0.651	0.75	1.19				
		***4***	148	0.99	0.902	0.78	1.25	1.001	0.854	0.990	1.012	154	0.99	0.941	0.79	1.25	1.001	0.794	0.991	1.013
	***M***	***2***	71	0.90	0.510	0.65	1.24					73	0.94	0.706	0.68	1.30				
		***3***	80	1.00	0.980	0.73	1.38					79	0.99	0.961	0.72	1.36				
		***4***	82	1.06	0.705	0.77	1.46	1.005	0.554	0.989	1.020	82	1.05	0.748	0.77	1.45	1.003	0.709	0.988	1.018
	***W***	***2***	76	1.06	0.725	0.76	1.48					70	0.95	0.784	0.68	1.33				
		***3***	72	1.01	0.977	0.72	1.40					68	0.90	0.550	0.65	1.26				
		***4***	66	0.91	0.587	0.64	1.29	0.997	0.743	0.982	1.013	72	0.93	0.672	0.67	1.30	1.000	0.986	0.984	1.016
***Cerebrovascular Diseases*** ***(430–438)***	***M + W***	***2***	385	1.03	0.699	0.89	1.19					387	1.06	0.456	0.91	1.23				
		***3***	403	1.12	0.129	0.97	1.29					394	1.11	0.178	0.96	1.28				
		***4***	399	1.06	0.476	0.91	1.22	1.004	0.240	0.997	1.011	407	1.05	0.480	0.91	1.22	1.004	0.307	0.997	1.010
	***M***	***2***	128	0.89	0.361	0.70	1.14					126	0.85	0.201	0.67	1.09				
		***3***	138	0.96	0.710	0.75	1.21					136	0.92	0.480	0.72	1.16				
		***4***	146	1.00	0.995	0.79	1.27	1.002	0.760	0.990	1.013	146	0.95	0.665	0.75	1.20	1.000	0.997	0.989	1.012
	***W***	***2***	257	1.11	0.260	0.92	1.34					261	1.20	0.059	0.99	1.45				
		***3***	265	1.23	0.028	1.02	1.48					258	1.24	0.025	1.03	1.49				
		***4***	253	1.10	0.332	0.91	1.33	1.006	0.199	0.997	1.014	261	1.13	0.192	0.94	1.37	1.006	0.191	0.997	1.014

Note—ICD-IX: International Classification of Diseases, IX revision; M: men; W: women; class exp.: class of noise exposure (see Table 1); 2: low noise exposure (night 43.7–49.5 dB (A); day 50.7–56.7 dB (A)); 3: medium noise exposure (night 49.5–53.3 dB (A); day 56.7–60.3 dB (A)); 4: high noise exposure (night 53.3–73.9 dB (A); day 60.3–78.1 dB (A)); *n*: numerosity; HR: hazard ratio; *p*: *p*-value; 95% CI: confidence interval at 95% of probability; Trend: risk trend for 1 decibel (dB (A)) noise increment. Adjustment factors: age classes, socio-economic deprivation index, and class of traffic pollution exposure.

**Table 4:** Hospitalization analysis (period 2001–2014) by period (nocturnal/diurnal), class of noise exposure, and gender.

Cause (Code ICD-IX)	Gender	ClassExp.	Nocturnal Period									Diurnal Period								
			*n*	HR	*p*	95% CI		Trend	*p*	95% CI		*n*	HR	*p*	95% CI		Trend	*p*	95% CI	
***Diseases of the Circulatory System*** ***(390–459)-CVD***	***M + W***	***2***	3345	1.00	0.959	0.95	1.05					3266	0.99	0.791	0.95	1.04				
		***3***	3364	1.01	0.808	0.96	1.06					3450	1.02	0.431	0.97	1.07				
		***4***	3319	1.02	0.423	0.97	1.07	1.002	0.137	0.999	1.004	3312	1.00	0.899	0.96	1.05	1.001	0.259	0.999	1.004
	***M***	***2***	1785	0.98	0.454	0.91	1.04					1745	0.97	0.384	0.91	1.04				
		***3***	1775	0.97	0.388	0.91	1.04					1862	1.01	0.840	0.94	1.08				
		***4***	1685	0.97	0.419	0.91	1.04	0.999	0.632	0.996	1.002	1649	0.94	0.082	0.88	1.01	0.999	0.448	0.996	1.002
	***W***	***2***	1560	1.03	0.454	0.96	1.10					1521	1.02	0.588	0.95	1.10				
		***3***	1589	1.05	0.163	0.98	1.13					1588	1.04	0.307	0.97	1.11				
		***4***	1634	1.08	0.033	1.01	1.16	1.005	0.006	1.001	1.008	1663	1.08	0.041	1.00	1.16	1.004	0.011	1.001	1.008
***Hypertensive Diseases*** ***(401–405)***	***M + W***	***2***	104	1.04	0.780	0.79	1.38					95	0.92	0.579	0.69	1.23				
		***3***	107	1.08	0.593	0.82	1.43					107	1.03	0.852	0.78	1.35				
		***4***	112	1.16	0.300	0.88	1.54	1.004	0.512	0.991	1.017	117	1.15	0.308	0.88	1.52	1.005	0.429	0.992	1.019
	***M***	***2***	54	1.19	0.408	0.79	1.77					49	1.06	0.779	0.71	1.59				
		***3***	51	1.13	0.557	0.75	1.70					54	1.14	0.512	0.77	1.70				
		***4***	48	1.10	0.672	0.72	1.66	1.003	0.763	0.984	1.022	49	1.08	0.707	0.72	1.64	1.004	0.661	0.985	1.024
	***W***	***2***	50	0.92	0.675	0.62	1.36					46	0.81	0.307	0.55	1.21				
		***3***	56	1.05	0.820	0.71	1.53					53	0.94	0.741	0.64	1.37				
		***4***	64	1.20	0.337	0.83	1.76	1.005	0.558	0.988	1.023	68	1.20	0.329	0.83	1.73	1.006	0.520	0.988	1.024
***Ischaemic Heart Diseases*** ***(410–414)-IHD***	***M + W***	***2***	923	0.94	0.194	0.86	1.03					886	0.91	0.034	0.83	0.99				
		***3***	970	1.00	0.977	0.91	1.09					1005	1.01	0.892	0.92	1.10				
		***4***	888	0.96	0.385	0.87	1.05	0.999	0.688	0.995	1.003	877	0.92	0.079	0.84	1.01	0.999	0.550	0.994	1.003
	***M***	***2***	608	0.93	0.232	0.84	1.05					576	0.88	0.025	0.78	0.98				
		***3***	619	0.96	0.483	0.86	1.07					651	0.98	0.662	0.87	1.09				
		***4***	542	0.90	0.090	0.80	1.02	0.996	0.196	0.991	1.002	533	0.86	0.014	0.77	0.97	0.996	0.137	0.990	1.001
	***W***	***2***	315	0.96	0.583	0.82	1.12					310	0.96	0.585	0.82	1.12				
		***3***	351	1.09	0.293	0.93	1.27					354	1.08	0.353	0.92	1.25				
		***4***	346	1.08	0.324	0.93	1.27	1.005	0.222	0.997	1.012	344	1.04	0.621	0.89	1.22	1.004	0.240	0.997	1.012
***Acute Myocardial Infarction*** ***(410)-AMI***	***M + W***	***2***	467	0.98	0.800	0.86	1.12					451	0.96	0.502	0.84	1.09				
		***3***	478	1.00	0.955	0.88	1.14					508	1.05	0.484	0.92	1.19				
		***4***	482	1.05	0.477	0.92	1.20	1.002	0.483	0.996	1.008	464	0.98	0.798	0.86	1.12	1.002	0.606	0.995	1.008
	***M***	***2***	298	0.95	0.565	0.81	1.12					278	0.89	0.154	0.76	1.05				
		***3***	296	0.94	0.463	0.80	1.11					330	1.02	0.765	0.88	1.20				
		***4***	290	0.98	0.798	0.83	1.15	0.999	0.741	0.991	1.006	274	0.90	0.225	0.76	1.07	0.998	0.620	0.990	1.006
	***W***	***2***	169	1.05	0.682	0.84	1.31					173	1.10	0.417	0.88	1.36				
		***3***	182	1.14	0.234	0.92	1.42					178	1.10	0.379	0.89	1.37				
		***4***	192	1.21	0.091	0.97	1.50	1.009	0.081	0.999	1.019	190	1.15	0.199	0.93	1.43	1.009	0.097	0.998	1.019
***Cerebrovascular Diseases*** ***(430–438)***	***M + W***	***2***	1078	0.98	0.565	0.90	1.06					1040	0.95	0.279	0.88	1.04				
		***3***	1087	0.99	0.790	0.91	1.08					1112	1.00	0.908	0.91	1.08				
		***4***	1083	0.99	0.861	0.91	1.08	1.002	0.451	0.998	1.006	1090	0.98	0.614	0.90	1.07	1.001	0.763	0.997	1.005
	***M***	***2***	501	0.92	0.157	0.81	1.04					483	0.89	0.058	0.78	1.00				
		***3***	525	0.96	0.514	0.85	1.09					545	0.97	0.605	0.86	1.09				
		***4***	486	0.92	0.183	0.81	1.04	0.997	0.374	0.992	1.003	481	0.89	0.064	0.78	1.01	0.996	0.220	0.990	1.002
	***W***	***2***	577	1.04	0.564	0.92	1.17					557	1.02	0.730	0.91	1.15				
		***3***	562	1.02	0.751	0.91	1.15					567	1.02	0.715	0.91	1.15				
		***4***	597	1.07	0.272	0.95	1.21	1.005	0.054	1.000	1.011	609	1.07	0.262	0.95	1.21	1.005	0.103	0.999	1.010
***Stroke*** ***(434,435,437,446)***	***M + W***	***2***	*603*	0.92	0.164	0.83	1.03					581	0.91	0.107	0.81	1.02				
		***3***	*626*	0.96	0.441	0.86	1.07					647	0.98	0.731	0.88	1.10				
		***4***	*608*	0.94	0.308	0.84	1.06	1.000	0.931	0.994	1.005	613	0.94	0.262	0.84	1.05	0.999	0.744	0.994	1.004
	***M***	***2***	*251*	0.87	0.102	0.73	1.03					247	0.87	0.115	0.73	1.03				
		***3***	*287*	0.98	0.785	0.83	1.15					296	1.00	0.991	0.85	1.18				
		***4***	*266*	0.95	0.529	0.80	1.12	0.999	0.824	0.991	1.007	266	0.94	0.476	0.79	1.12	0.999	0.757	0.991	1.007
	***W***	***2***	*352*	0.97	0.679	0.83	1.13					334	0.94	0.457	0.81	1.10				
		***3***	*339*	0.94	0.446	0.81	1.10					351	0.97	0.650	0.83	1.12				
		***4***	*342*	0.94	0.431	0.81	1.10	1.000	0.934	0.993	1.007	347	0.94	0.394	0.80	1.09	0.999	0.878	0.992	1.007

Note—ICD-IX: International Classification of Diseases, IX revision; M: men; W: women; class exp.: class of noise exposure (see Table 1); 2: low noise exposure (night 43.7–49.5 dB (A); day 50.7–56.7 dB (A)); 3: medium noise exposure (night 49.5–53.3 dB (A); day 56.7–60.3 dB (A)); 4: high noise exposure (night 53.3–73.9 dB (A); day 60.3–78.1 dB (A)); *n*: numerosity; HR: hazard ratio; *p*: *p*-value; 95% CI: confidence interval at 95% of probability; Trend: risk trend for 1 decibel (dB (A)) noise increment. Adjustment factors: age classes, socio-economic deprivation index and class of traffic pollution exposure.

**Table 5:** Synoptic table summarizing the results of the statistical analyses.

MORTALITY (Period 2001–2012)						
Cause (Code ICD-IX)	Gender	Class of Exposure	Night		Day	
			+/-	Trend	+/-	Trend
***Diseases of the Circulatory System*** ***(390–459)-CVD***	***M + W***	***2***				
		***3***	+		+	
		***4***	+	t	+	t
	***W***	***2***				
		***3***	+		+	
		***4***	+	t	+	t
***Ischaemic Heart Diseases*** ***(410–414)-IHD***	***W***	***2***				
		***3***				
		***4***		t		t
***Cerebrovascular Diseases*** ***(430–438)***	***W***	***2***				
		***3***	+		+	
		***4***				
**HOSPITALIZATION (Period 2001–2014)**						
**Cause (Code ICD-IX)**	**Sex**	**Class of Exposure**	**Night**		**Day**	
			**+/-**	**Trend**	**+/-**	**Trend**
***Diseases of the Circulatory System*** ***(390–459)-CVD***	***M***	***2***				
		***3***				
		***4***			-	
	***W***	***2***				
		***3***				
		***4***	+	t	+	t
***Ischaemic Heart Diseases*** ***(410–414)-IHD***	***M + W***	***2***				
		***3***				
		***4***			-	
	***M***	***2***				
		***3***				
		***4***	-		-	
***Acute Myocardial Infarction*** ***(410)-AMI***	***W***	***2***				
		***3***				
		***4***	+	t		t
***Cerebrovascular Diseases*** ***(430–438)***	***M***	***2***				
		***3***				
		***4***			-	
	***W***	***2***				
		***3***				
		***4***		t		

Note—ICD-IX: International Classification of Diseases, IX revision; M: men; W: women; 2: low noise exposure (night 43.7–49.5 dB (A); day 50.7–56.7 dB (A)); 3: medium noise exposure (night 49.5–53.3 dB (A); day 56.7–60.3 dB (A)); 4: high noise exposure (night 53.3–73.9 dB (A); day 60.3–78.1 dB (A)); +: risk excess; -: risk defect; trend: risk trend for 1 decibel (dB (A)) noise increment; t: risk trend. The threshold for statistical significance was set at *p* < 0.05 but associations with *p* < 0.10 were also reported.
